# No evidence for effects of low-intensity vestibular noise stimulation on mild-to-moderate gait impairments in patients with Parkinson’s disease

**DOI:** 10.1007/s00415-024-12504-z

**Published:** 2024-06-17

**Authors:** Daniela Peto, Florian Schmidmeier, Sabrina Katzdobler, Urban M. Fietzek, Johannes Levin, Max Wuehr, Andreas Zwergal

**Affiliations:** 1 German Center for Vertigo and Balance Disorders (DSGZ), LMU University Hospital, Ludwig-Maximilians-Universität München, Marchioninistrasse 15, 81377 Munich, Germany; 2 Department of Neurology, LMU University Hospital, LMU Munich, Munich, Germany; 3 Deutsches Zentrum für Neurodegenerative Erkrankungen (DZNE) e.V., Munich, Germany; 4 Munich Cluster of Systems Neurology (SyNergy), Munich, Germany; 5 Schön Klinik München Schwabing, Munich, Germany

**Keywords:** Parkinson’s disease, Gait disorder, Locomotion, Galvanic vestibular stimulation, Stochastic resonance

## Abstract

**Background:**

Gait impairment is a key feature in later stages of Parkinson’s disease (PD), which often responds poorly to pharmacological therapies. Neuromodulatory treatment by low-intensity noisy galvanic vestibular stimulation (nGVS) has indicated positive effects on postural instability in PD, which may possibly be conveyed to improvement of dynamic gait dysfunction.

**Objective:**

To investigate the effects of individually tuned nGVS on normal and cognitively challenged walking in PD patients with mild-to-moderate gait dysfunction.

**Methods:**

Effects of nGVS of varying intensities (0–0.7 mA) on body sway were examined in 32 patients with PD (ON medication state, Hoehn and Yahr: 2.3 ± 0.5), who were standing with eyes closed on a posturographic force plate. Treatment response and optimal nGVS stimulation intensity were determined on an individual patient level. In a second step, the effects of optimal nGVS vs. sham treatment on walking with preferred speed and with a cognitive dual task were investigated by assessment of spatiotemporal gait parameters on a pressure-sensitive gait carpet.

**Results:**

Evaluation of individual balance responses yielded that 59% of patients displayed a beneficial balance response to nGVS treatment with an average optimal improvement of 23%. However, optimal nGVS had no effects on gait parameters neither for the normal nor the cognitively challenged walking condition compared to sham stimulation irrespective of the nGVS responder status.

**Conclusions:**

Low-intensity nGVS seems to have differential treatment effects on static postural imbalance and continuous gait dysfunction in PD, which could be explained by a selective modulation of midbrain-thalamic circuits of balance control.

## Introduction

Gait impairments are among the most disabling symptoms in patients with Parkinson’s disease (PD), which lead to reduced mobility and recurrent falls [14, 32]. Locomotor disturbances aggravate as the disease progresses, and significantly compromise social independence and quality of life. The widespread impact of PD pathology on the structure and function of the supraspinal locomotor network [20, 27] determines the phenotype of parkinsonian gait, characterized by a reduced range and speed of movement, impaired symmetry and rhythmicity of stepping, and compromised balance and postural control [15, 21, 36]. Besides these continuous alterations of walking, parkinsonian gait is linked to episodic locomotor disturbances such as festination and freezing of gait [17, 32, 36].

Standard pharmacological therapy in PD with dopaminergic medication can improve certain aspects of parkinsonian gait, in particular the speed and amplitude of locomotor movements [15, 42]. However, motor fluctuations and dyskinesia that often accompany prolonged dopaminergic therapy may conversely induce detrimental effects on gait and postural regulation and result in an increased risk of falling [11, 22, 40]. Analogously, deep brain stimulation of the subthalamic nucleus or globus pallidus—a prevailing treatment strategy in later stages of disease—may induce moderate improvements in gait and static balance, but appears to be less effective or even detrimental to dynamic postural stability [44, 46, 57].

Numerous non-invasive brain stimulation methods are currently being tested with the aim of closing the therapeutic gap for PD-associated (loco-)motor symptoms that do not adequately respond to available treatment options. Among these, galvanic vestibular stimulation (GVS)—a non-invasive technique to modulate the activity of vestibular afferents [13]—has gained special interest due to the close anatomic and functional connection between central vestibular networks and brain structures affected by PD pathology [29]. Administered as zero-mean low-intensity white noise current, noisy GVS (nGVS) has been shown to sensitize peripheral vestibular afferent activity and improve central vestibular sensorimotor function (for review see [13, 50]). In patients with PD and atypical Parkinsonism, nGVS has been successfully administered to reduce postural imbalance and improve other PD-associated motor and autonomic symptoms [30, 38, 55, 56, 58]. The presumed mechanism of these therapeutic effects is stochastic resonance—a phenomenon where the response of a sensory system to weak, sub-threshold signals can be enhanced by a small amount of noise [10, 31].

The therapeutic effect of nGVS on postural instability—a core element of parkinsonian gait—suggests that low-intensity vestibular noise stimulation might also positively impact gait disturbance in afflicted patients. This assumption is further supported by animal experiments that demonstrate that nGVS can alleviate locomotor dysfunction in a parkinsonian rodent model [38]. In this light, the aim of this study was to examine this presumed therapeutic effect of nGVS on locomotor function in patients with PD. Since nGVS treatment response is known to critically depend on the applied stimulation intensity that may differ between individual patients, we initially determined optimal treatment response parameters in individual patients using an established static balance task [56]. In a second step, we studied the effects of optimal nGVS vs. sham treatment on normal and cognitively challenged walking in a larger cohort of patients with PD by using a comprehensive assessment of spatiotemporal walking characteristics.

## Materials and methods

### Participants

Thirty-two patients with PD (age 68.1 ± 11.3 years, 7 females) participated in this study. Prior to experimental testing, each patient underwent a complete physical, neurological and neuro-otological examination by an expert neurologist (AZ) including an assessment of gait impairment and functional mobility by the Functional Gait Assessment (FGA) [48]. Clinical scoring of disease stage and symptom severity (Hoehn and Yahr Scale, H&Y; Movement Disorders Society-Unified Parkinson’s Disease Rating Scale, MDS-UPDRS) revealed mild-to-moderate disease severity (H&Y of 2.3 ± 0.5 and MDS-UPDRS of 34.6 ± 22.3). Based on the MDS-UPDRS outcomes, patients were, where appropriate, categorized into the two different subtypes, i.e., tremor dominant (TD) and postural instability and gait difficulty (PIGD) [23]. Accordingly, 23 had a PIGD subtype (72%), 6 had a TD subtype (19%), and 3 could not be assigned to either subtype. In addition, the Beck Depression Inventory (BDI) and the Montreal Cognitive Assessment (MOCA) were performed to rate patients’ non-motor symptoms such as depression and cognitive impairment. None of the patients did show any signs of atypical parkinsonism, relevant auditory or manifest vestibular disorders. l-DOPA was the basic medication in all patients (mean daily dose: 719 ± 357 mg). Regular medication was continued during study participation. All participants gave written informed consent prior to study inclusion.

### Galvanic vestibular stimulation

Vestibular noise stimulation (i.e., nGVS) was applied via a pair of 4.0 cm × 6.0 cm Ag–AgCl electrodes attached bilaterally over the left and right mastoid process. Zero-mean Gaussian white noise stimulation with a frequency range of 0–30 Hz and varying peak amplitudes of 0–0.7 mA was delivered by a mobile constant current stimulator (neuroConn^®^, Illmenau, Germany).

### Experimental procedures

The experimental procedures consisted of two parts: in a first step, the nGVS response and stimulation intensity that optimally stabilized posture was determined in each patient using static posturography. In the main part, the effects of nGVS at individually optimal stimulation intensity on gait performance was assessed in comparison to sham stimulation (i.e., nGVS at 0 mA).

**Fig. 1:**
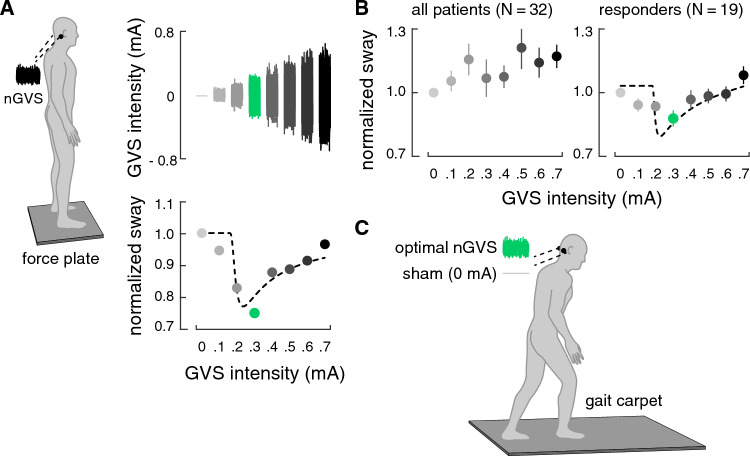
Experimental setup and procedures. **A** Effects of noisy galvanic vestibular stimulation (nGVS) on static balance in patients were initially determined on a posturographic force plate (left side). Right side: exemplary modulation of body sway (simulated data, lower panel) across the administered nGVS intensities (upper panel) that follows a bell-shaped performance curve indicative of the presence of stochastic resonance (SR; model fit: dashed line). The green filled dot indicates the optimal reduction of body sway at a particular nGVS level. **B** Group average nGVS on static balance across all patients (left side). Right side: 19 of in total 32 patients exhibited an SR-like response curve to stimulation with optimal balance improvements at intermediate noise intensities. **C** Subsequently, the individually optimal nGVS intensity in responding patients and a default intensity of 0.3 in non-responding patients was used to evaluate nGVS effects vs. sham stimulation (nGVS at 0 mA) on walking performance on a pressure-sensitive gait carpet

The initial evaluation of nGVS on static posture was performed according to previously established procedures [3, 56]. Body sway was recorded for 30 s on a force plate (Kistler, 9261A, Kistler Group, Winterthur, Switzerland) at 40 Hz while standing with eyes closed (Fig. 1A). This procedure was repeated eight times, while patients were stimulated with a different amplitude of nGVS (ranging from 0 to 0.7 mA, in a randomized order) in each trial. Patients were blinded to the stimulation order. For each stance trial, mean sway velocity [mm/s] was calculated as the primary output measures based on the recorded radial center-of-pressure (CoP) trajectory. Parameters from the 8 stance trials were normalized to sway velocity obtained during 0 mA stimulation (i.e., baseline condition).

To determine the presence or absence of a treatment response of nGVS on static balance, a theoretical SR-curve was fit on the normalized balance responses data using a goodness-of-fit statistic [3, 16] (Fig. 1A). Three experienced human raters (i.e., MW, DP, and AZ) subsequently independently evaluated whether balance responses to nGVS were compatible with a bell-shaped response curve with improvements of performance at intermediate stimulation intensities based on a visual inspection of the individual body sway modulations and the corresponding SR-curve fit. Patients for which all raters identified a treatment response were designated as “responders” and the nGVS intensities that optimally stabilized posture was taken for further evaluation of nGVS effects on walking performance. The remaining patients were designated as “non-responders” and a fixed nGVS level of 0.3 mA that corresponds to the average optimal nGVS intensity from previous reports in patients from different clinical cohorts (including PD) [16, 25, 52, 53, 55, 56] was taken for assessment of treatment responses on gait.

In the main part of the experiment, nGVS effects on gait were assessed during walking at preferred speed and during walking with a cognitive dual task (DTC: verbal fluency, i.e., naming items of a given category)—a condition that is known to exacerbate gait impairments in patients with PD. Both conditions were assessed during stimulation with optimal nGVS intensity (see above) and during sham stimulation (i.e., nGVS at 0 mA) in a pseudo-randomized order with patients blinded to the stimulation condition (Fig. 1C). Walking performance was evaluated on a 6.7 m long pressure-sensitive gait carpet (GAITRite^®^, CIR Systems, Havertown, USA) with a sampling frequency of 120 Hz. For each gait condition, patients performed in total four repetitions to collect enough strides for further analysis. Gait performance was characterized based on five established gait metrics that represent the five primary gait domains previously established for parkinsonian gait [15]: gait velocity, swing phase, stride time variability (computed by the coefficient of variation, CV), stride time asymmetry, and base of support. Dual task walking performance was quantified by calculating dual task costs for each gait metric, i.e., the percentage change of performance during dual task walking compared to single task walking (ST: preferred walking): ${\text{DTC}}_{\text{COST}}= 100 \times ({\text{metric}}_{\text{DTC}}-{\text{metric}}_{\text{ST}})/{\text{metric}}_{\text{ST}}$. In an analogous manner, cognitive performance during dual task walking (i.e., item count per second) was compared to cognitive single task performance (i.e., item count per second while sitting) that was examined before the start of the gait assessment. Upon completion of the gait assessment with nGVS and sham stimulation, patients were asked whether they experienced any change in gait balance (categories: no change, improvement, deterioration).

### Statistical analysis

Stimulation effects on gait performance (nGVS vs. sham) were evaluated using a Wilcoxon matched-pairs signed-rank test with Bonferroni correction for multiple comparisons, because not all gait metrics were found to be distributed normally. This comparison was performed once for all patients and in addition for the subgroups of “responding” and “non-responding” patients as designated after the assessment of nGVS effects of static balance. Results were considered significant at *p* < 0.05. Statistical analysis was performed using SPSS (Version 26.0, IBM Corp., USA).

### Data availability

Data reported in the article will be shared by any appropriately qualified investigator on request after pseudonymization.

## Results

In a first part of the experiment, stimulation effects on static balance across varying nGVS intensities ranging from 0 to 0.7 mA were assessed. Administration of nGVS was well tolerated and did not cause disequilibrium in any of the examined patients. Evaluation of individual balance responses yielded that 19 out of 32 patients (59%) displayed a beneficial balance response to nGVS treatment indicated by a bell-shaped performance curve with optimal improvements of balance at intermediate stimulation intensities (Fig. 1B). These patients were designated as “responders” with an average optimal improvement of 23% (range 3 to 49%) at an average intensity of 0.3 mA (range: 0.1 to 0.5 mA). An analysis regarding the different PD subtypes further revealed that 65% of the PIGD patients responded positively compared to only 33% of the TD patients. In the remaining patients, which were designated as “non-responders”, body sway velocity either randomly fluctuated or was generally increased across the range of tested nGVS intensities.

**Fig. 2:**
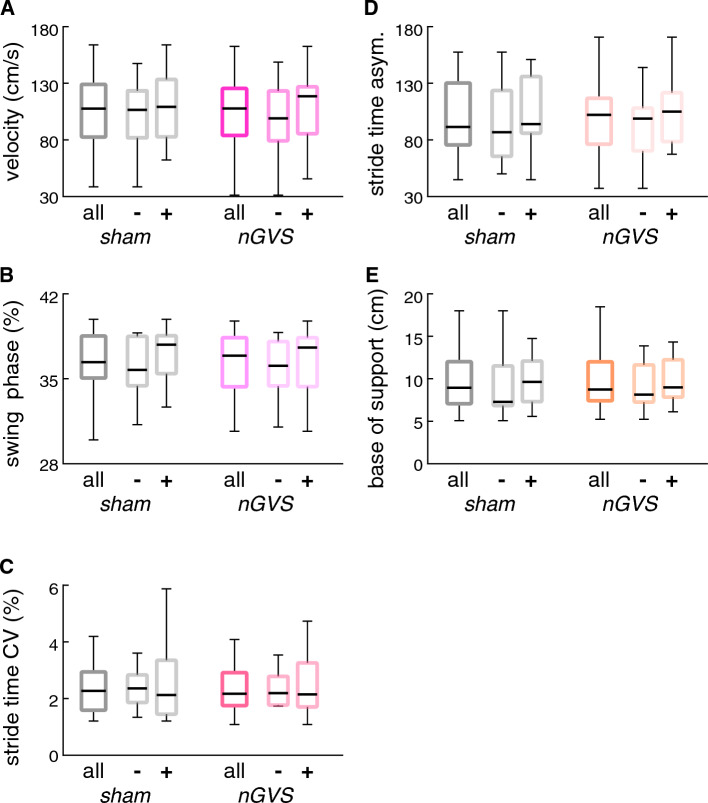
Stimulation effects on preferred walking. Effects of nGVS at individually optimal vs. sham stimulation (nGVS intensity at 0 mA) on gait performance during preferred walking. Results are displayed for all patients as well as subdivided for patients that did (responders: +) vs. did not (non-responders: **−**) show improvement of static balance during nGVS treatment. *CV* coefficient of variation, *asym* asymmetry

**Fig. 3:**
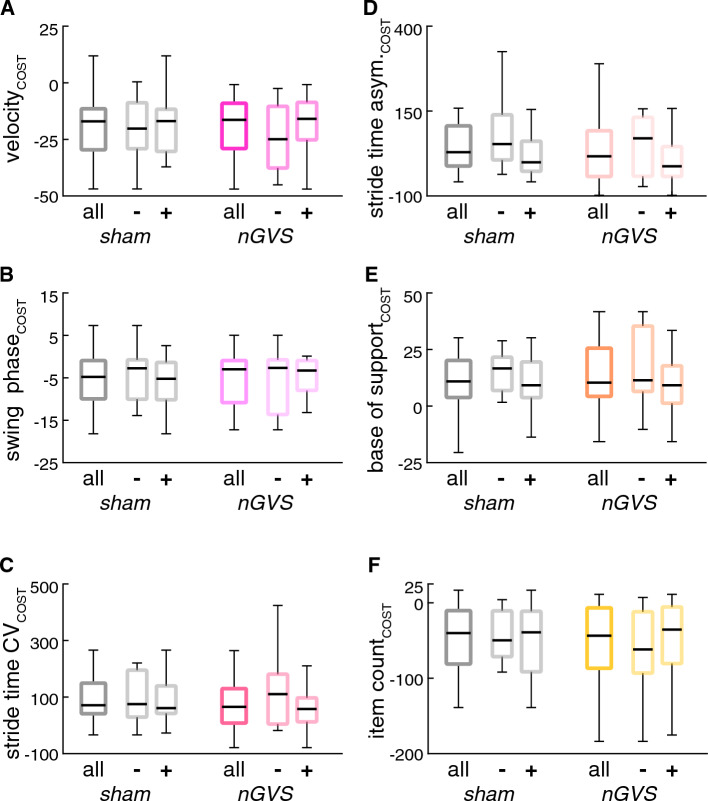
Stimulation effects on walking with cognitive dual task. Effects of nGVS at individually optimal vs. sham stimulation (nGVS intensity at 0 mA) during walking with a cognitive dual task (verbal fluency) on gait (**A**–**E**) and concomitant cognitive performance (**F**). Performance metrics are displayed as dual task cost, i.e., percentage change of performance during dual task in relation to performance during preferred walking (gait metrics) or cognitive single task (cognitive metric). Results are displayed for all patients as well as subdivided for patients that did (responders: +) vs. did not (non-responders: **−**) show improvement of static balance during nGVS treatment. *CV* coefficient of variation, *asym* asymmetry

In a second step, nGVS effects on gait performance were examined. Baseline assessment of gait and functional mobility by the FGA revealed mild-to-moderate gait impairments (FGA: 24.0 ± 5.8/30) with only 4 patients meeting the criterion of FGA ≤ 15/30, indicative for an increased risk of falling in PD [28]. Stimulation effects at individually optimal nGVS intensity (non-responders received a fixed nGVS level of 0.3 mA) during instrumented gait assessment with preferred (Fig. 2) and cognitively challenged walking (Fig. 3) were compared to sham stimulation (i.e., nGVS at 0 mA). nGVS did not have any effect on walking performance, neither in the entire patient cohort nor when focusing only on patients that showed a beneficial stimulation effect on static balance (i.e., responders). Analogously, cognitive performance during dual task walking was found to be unaltered during nGVS compared to sham stimulation. These observations were also reflected in patients’ subjective rating of gait balance between conditions: most patients (94%) did not report any difference in balance, whereas two patients reported a moderate deterioration of walking balance during stimulation.

## Discussion

Previous reports repeatedly demonstrated that vestibular noise stimulation administered at weak sub-threshold intensities (i.e., nGVS) can alleviate static postural symptoms in patients with PD [34, 38, 56, 58]. Based on these findings, we hypothesized that nGVS may also induce improvements in parkinsonian gait deficits that are among others characterized by an impaired dynamic balance control. In this light, we systematically investigated whether therapeutic effects of nGVS on PD-related deficits in static balance regulation may expand to improvements in PD-associated locomotor deficits. However, while we could confirm a robust nGVS treatment response on balance regulation in nearly two thirds of the assessed patients [56], we did not observe any systematic therapeutic effect of nGVS on locomotor performance. This negative finding was consistent across patients that did or did not exhibit nGVS-induced improvements on static balance deficits and for normal walking as well as walking during cognitive dual task—a condition that typically aggravates locomotor deficits in parkinsonian gait. At first sight, our observations seem to be inconsistent with a previous report on nGVS-induced improvements in locomotor dysfunction in a parkinsonian rodent model [38]. On closer examination, however, the performance metric assessed in this study, i.e., the rotarod test, rather reflects motor coordination and balance regulation than steady-state locomotor function [9]. Hence, there seems to be a general discrepancy between the positive response of postural symptoms to nGVS therapy and the absence of treatment effects on PD-related locomotor dysfunction. In the following, we will discuss potential reasons for this discrepancy and consider methodological limitations that may have influenced this negative outcome.

Previous literature suggests that nGVS may unfold treatment responses on balance and locomotor performance in patients with PD along at least two different pathways. nGVS-induced stochastic resonance at the vestibular periphery not only sensitizes vestibular perception per se [16, 25, 26, 52] but has further been demonstrated to augment the responsiveness of balance-related vestibulospinal reflexes [39, 49]. Deficits within these central vestibular sensorimotor pathways have been assumed to contribute to balance and locomotor deficits in patients with PD (for a review see [41]). Hence, nGVS may exhibit a direct descending therapeutic influence on impaired vestibulospinal balance reflexes in patients with PD. However, while improved vestibulospinal reflex responses in healthy individuals and patients with peripheral vestibular hypofunction have been linked to behaviorally relevant improvements of both static balance regulation and locomotion performance [18, 53, 54], nGVS in patients with PD appears to primarily affect static balance regulation but not locomotion. nGVS-induced improvements along descending vestibulospinal pathways, therefore, most likely do not account for the observed discrepancy between treatment responses of balance and locomotor dysfunction in PD.

Alternatively, nGVS treatment in PD could act along ascending pathways that connect vestibular afferents to the thalamus and basal ganglia [8, 43]. Recent studies in rodents demonstrate that gait parameters such as initiation, speed or direction are encoded in distinct basal ganglia-brainstem circuits, which are defined by a granular in- and output connectivity and neurotransmitter release [2]. High-speed locomotion for instance is mostly controlled by an excitatory glutamatergic projection from the cuneiform nucleus within the mesencephalic locomotor region (MLR) to the lateral paragigantocellular nucleus in the medulla [7, 24]. Dopaminergic neurons from substantia nigra pars compacta do not convey information about the movement pattern but seem to modulate its initiation and vigor [12]. Another subdivision of the MLR, the pedunculopontine nucleus (PPN), provides primary cholinergic input to the thalamus, which has been implicated in postural control. Vestibular input directly modulates PPN activity [1] and in line with this nGVS has been previously shown to influence PPN connectivity in patients with PD [8]. Imbalance and gait disturbance in PD are generally linked to alterations in cholinergic input but appear to involve topographically distinct cholinergic networks. While parkinsonian gait deficits including freezing of gait have been predominantly linked to cholinergic deficits in the basal forebrain nuclei [33, 47], postural instability and falls appear to be primarily linked to cholinergic input from the PPN [4–6, 37]. Hence, nGVS-induced effects along ascending PPN-thalamo-cortical connections appear to be well compatible with a primary treatment response of postural symptoms and a weak to absent response in PD-related gait deficits [30] and would thus support the observed discrepancy between treatment responses of PD-related balance and locomotor symptoms to nGVS.

The current observations must be interpreted with respect to certain methodological limitations. First, nGVS treatment responses are known to depend on the applied stimulation intensity that may differ between individual patients. Since our comprehensive and lengthy gait assessment procedure precluded an evaluation of treatment effects across varying stimulation intensities, we limited our examination to the nGVS intensity that optimally improved posture in the initial static balance task. We can therefore not rule out the possibility that an application of lower or higher nGVS intensities might have conversely resulted in observable locomotor improvements. Previous reports from healthy individuals and patients with peripheral vestibular hypofunction suggest, however, that nGVS improves static balance and locomotion at intensities that are largely consistent [18, 19, 51, 53, 54]. Secondly, our current gait assessment specifically focused on continuous aspects of parkinsonian gait deficits, i.e., spatiotemporal alterations during steady-state walking. It is, however, conceivable that nGVS may ameliorate other not examined aspects of parkinsonian gait, in particular intermittent locomotor disturbances such as festination and freezing of gait. Thirdly, in accordance with previous studies [34, 35, 45, 58], we examined nGVS effects on balance and locomotor function in patients’ ON motor state. This could have led to the masking of potentially present, moderate treatment effects of nGVS on locomotor function by the dopaminergic medication effects. However, in a future treatment scenario, nGVS would not be applied as a substitute but rather as a supplement to established pharmacological treatment options (e.g., l-DOPA). Finally, our study cohort included primarily patients at mild-to-moderate disease stages (H&Y: 2.3 ± 0.5), with moderate locomotor deficits. With respect to balance responses, previous reports suggest that nGVS treatment is particularly effective in patients at later stages of disease [56]. Hence, follow-up studies are required to investigate potential therapeutic effects of low-intensity vestibular noise stimulation on continuous and episodic aspects of parkinsonian gait in particular in patients at later stages of PD [8].
